# Efficacy and safety of programmed cell death protein-1 inhibitor for first-line therapy of advanced gastric or gastroesophageal junction cancer: a network meta-analysis

**DOI:** 10.3389/fimmu.2025.1500954

**Published:** 2025-04-08

**Authors:** Yunnan Zhang, Wenxing Peng, Wei Yang, Wenzhou Zhang, Yannan Fan

**Affiliations:** ^1^ Department of Pharmacy, The Affiliated Cancer Hospital of Zhengzhou University and Henan Cancer Hospital, Zhengzhou, China; ^2^ Department of Pharmacy, Beijing Anzhen Hospital, Capital Medical University, Beijing, China

**Keywords:** advanced gastric cancer, gastroesophageal junction cancer, first-line treatment, PD-1 inhibitor, network meta-analysis

## Abstract

**Background:**

This study conducted a network meta-analysis to evaluate and rank the safety and efficacy of programmed cell death protein-1 (PD-1) inhibitors for patients with advanced gastric or gastroesophageal junction cancer (GC/GEJC).

**Methods:**

A systematic search was conducted in PubMed, Embase, and Cochrane Library databases to compare the efficacy and safety of different treatment regimens, including overall survival (OS), progression-free survival (PFS), objective response rate (ORR), and treatment-related adverse events (TRAEs) in patients with advanced GC/GEJC.

**Results:**

A total of six RCT studies were ultimately included in the analysis, involving 6,294 patients. Among them, 256 patients received PD-1 inhibitor monotherapy (pembrolizumab), 3,029 patients received a PD-1 inhibitor plus chemotherapy (1,047 with pembrolizumab, 1,154 with nivolumab, 327 with sintilimab, and 501 with tislelizumab), and 3,009 received either chemotherapy or chemotherapy plus placebo. Sintilimab plus chemotherapy had the highest SUCRA value for OS (85.2%), while nivolumab plus chemotherapy had the highest SUCRA values for both PFS and ORR (96.8% and 82.9%). Four PD-1 inhibitors plus chemotherapy significantly improved median OS and ORR compared with chemotherapy. Sintilimab plus chemotherapy, pembrolizumab plus chemotherapy, and nivolumab plus chemotherapy significantly improved median PFS compared with chemotherapy. For TRAEs of grade 3 or worse, pembrolizumab monotherapy had the highest SUCRA value. Tislelizumab plus chemotherapy, as well as sintilimab plus chemotherapy, did not increase the overall incidence of TRAEs and the incidence of grade 3 or worse TRAEs.

**Conclusions:**

In the first-line treatment of advanced GC/GEJC, PD-1 inhibitors plus chemotherapy have been demonstrated to significantly improve OS, PFS, and ORR compared with chemotherapy. Among them, sintilimab plus chemotherapy achieved the highest SUCRA value for OS, and nivolumab plus chemotherapy achieved the highest SUCRA values for PFS and ORR. Regarding safety, tislelizumab plus chemotherapy and sintilimab plus chemotherapy did not increase the overall incidence of TRAEs and the incidence of grade 3 or worse TRAEs, with good tolerability and safety.

## Introduction

Gastric cancer (GC) is one of the malignant tumors with high morbidity and mortality rates worldwide. According to the GLOBOCAN data in 2020, there are more than one million new cases of GC every year globally, with approximately 760,000 deaths (1). The incidence is particularly high in East Asia (2). Due to the asymptomatic or minimally symptomatic presentation in the early stage of GC, approximately 80% of patients with GC are unfortunately diagnosed at locally advanced or terminal stages, making surgical intervention difficult to achieve radical cure. Furthermore, GC cells exhibit poor sensitivity to chemotherapy agents and may carry drug resistance genes, resulting in a poor prognosis and shorter survival times for patients with advanced gastric cancer undergoing systemic therapy (3). Consequently, there is still a lack of effective treatment options for the patient population.

Recently, immunotherapy has made breakthrough progress in the treatment of GC (4). Immunotherapy focuses on identifying and attacking tumor cells by activating or enhancing the patient’s immune system, the most representative of which are programmed death protein 1 (PD-1) and programmed death ligand 1 (PD-L1) inhibitors (5). PD-1/L1 inhibitors have demonstrated significant efficacy in some patients with advanced GC by blocking the interaction between PD-1 and PD-L1, thereby reactivating the anti-tumor activity of T cells and enhancing the immune response of the body. Multiple studies have confirmed the efficacy and safety of several PD-1 inhibitors in the treatment of advanced GC, and these studies provide further support for PD-1 inhibitors as the first-line treatment in advanced GC (4, 6, 7). However, there remains a deficiency in conducting head-to-head comparisons among different treatment regimens. Network meta-analysis provides a suitable methodology for cancer research to evaluate and rank the efficacy of various treatment options through direct and indirect evidence. This study conducted a network meta-analysis to evaluate and rank the safety and efficacy of different treatment regimens for untreated advanced GC, specifically comparing PD-1 inhibitor monotherapy, chemotherapy, and PD-1 inhibitors plus chemotherapy. The findings of this analysis provide evidence-based support for future treatment options.

## Methods

### Search strategy

A systematic search was conducted in PubMed, Embase, and Cochrane Library databases using Medical Subject Headings (MeSH) terms and free text to screen for clinical trials on advanced GC or gastroesophageal junction cancer (GEJC). The search period spanned from the inception of the database to June 11, 2024. The complete search strategy is presented in **Supplementary Table 1**. The review protocol was registered in the International Prospective Register of Systematic Reviews (PROSPERO) (CRD42024584758).

### Selection criteria

Two reviewers (Y. Zhang and W. Peng) independently screened original studies by reviewing titles, abstracts, and full texts. The third reviewer (Y. Fan) resolved their disagreements. Studies that met the following inclusion criteria were included: (1) The study population comprised patients with advanced GC/GEJC; (2) The studies investigated PD-1 inhibitor monotherapy or its combination with chemotherapy as first-line treatment, in comparison to chemotherapy.

Studies were excluded if they belonged to any of the following categories: (1) Non-randomized controlled trials (RCTs); (2) Conference abstracts; (3) Publications not in English; (4) Studies that did not report any of the outcome measures, including overall survival (OS), progression-free survival (PFS), objective response rate (ORR), treatment-related adverse events (TRAEs), and immune-related adverse events (irAEs).

### Data extraction and risk of bias assessment

Data extraction was performed independently by two researchers (Y. Zhang and W. Peng). Basic information was collected, including title, first author, publication year, patients’ age, clinical trial phase, sample size, median follow-up duration, intervention in the experimental and control groups, and outcome measures (including OS, PFS, ORR, TRAEs and irAEs).

The Cochrane Collaboration’s tool for assessing risk of bias was applied independently to evaluate literature quality. Risk of bias assessments included random sequence generation, allocation concealment, blinding of participants and personnel, blinding of outcome assessment, incomplete outcome data (rated as low risk if intention-to-treat analysis was utilized), selective reporting, and other biases (including baseline comparability between the experimental and control groups, and adherence to the trial protocol). Each risk can be rated as low, moderate, high, or unclear risk.

### Statistical analysis

All statistical analyses were conducted using Stata 15 software. Residual analysis was performed to assess global consistency by comparing the differences between the “consistency” and “inconsistency” models. The I^2^ statistic was applied to evaluate the overall heterogeneity, with I^2^ values of <25%, 25%-50%, and >50% indicating low, moderate, or high inconsistency, respectively. When I^2^ > 50%, a random-effects model was adopted; otherwise, a fixed-effects model was used. The Surface Under the Cumulative Ranking (SUCRA) represents the cumulative ranking probabilities, indicating the probability that a treatment ranks higher among all available treatments. When the SUCRA value is 1, the treatment is the best, whereas when the SUCRA value is 0, the treatment is the worst (8). Odds ratios (ORs) with 95% confidence intervals (CIs) were used as the pooled effect size to evaluate categorical variables, while mean differences with 95% CIs were adopted for continuous variables.

Due to the presence of many indirect comparisons in the network meta-analysis, this may pose challenges in drawing appropriate conclusions. To further strengthen the results, both SUCRA and point estimate results should be considered when determining the optimal treatment strategy, with point estimates using chemotherapy as the reference (since it is more closely connected to other treatments). In addition, the consistency between point estimates and SUCRA values should be checked.

## Results

### Literature search

**Figure 1:**
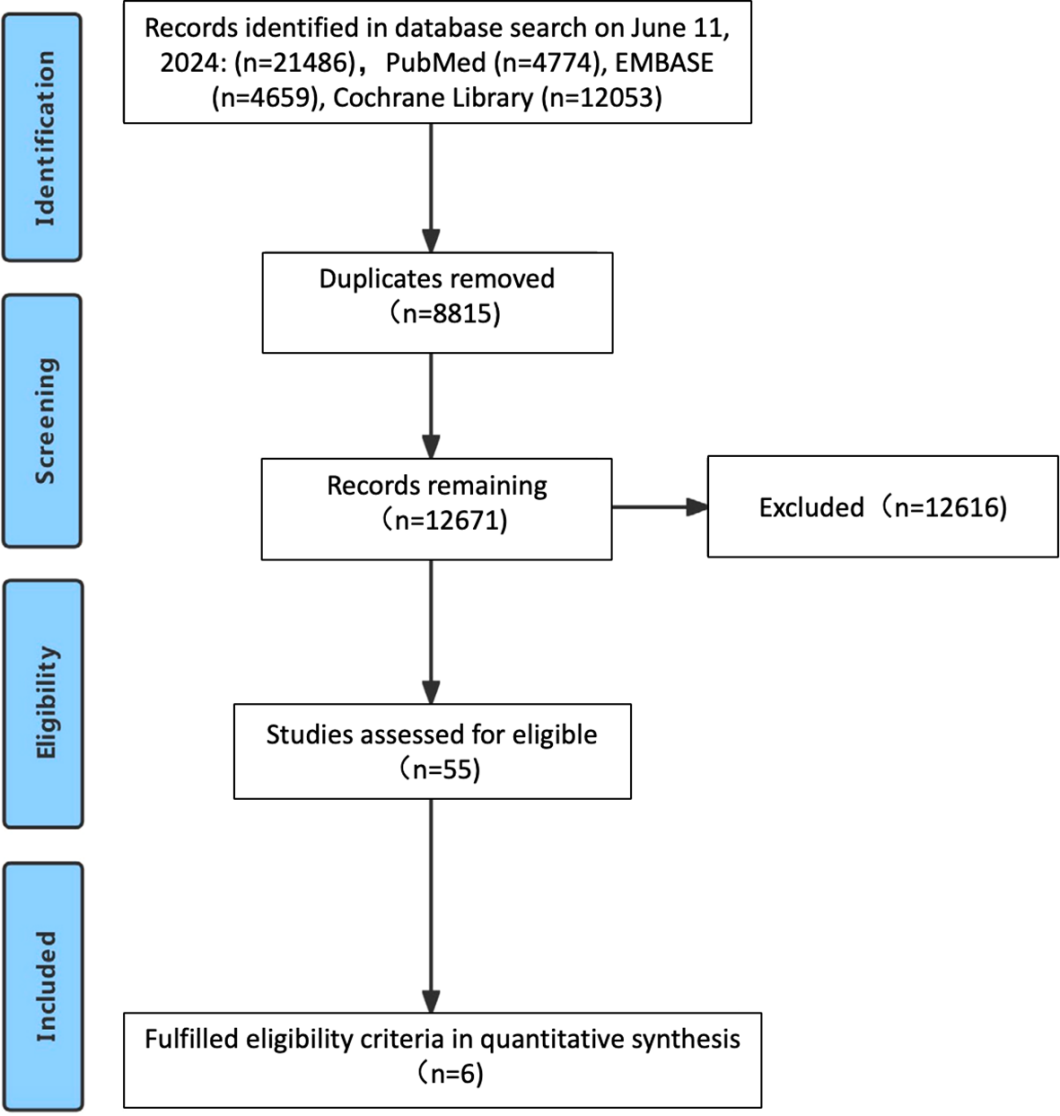
Flowchart of study selection.

**Figure 2:**
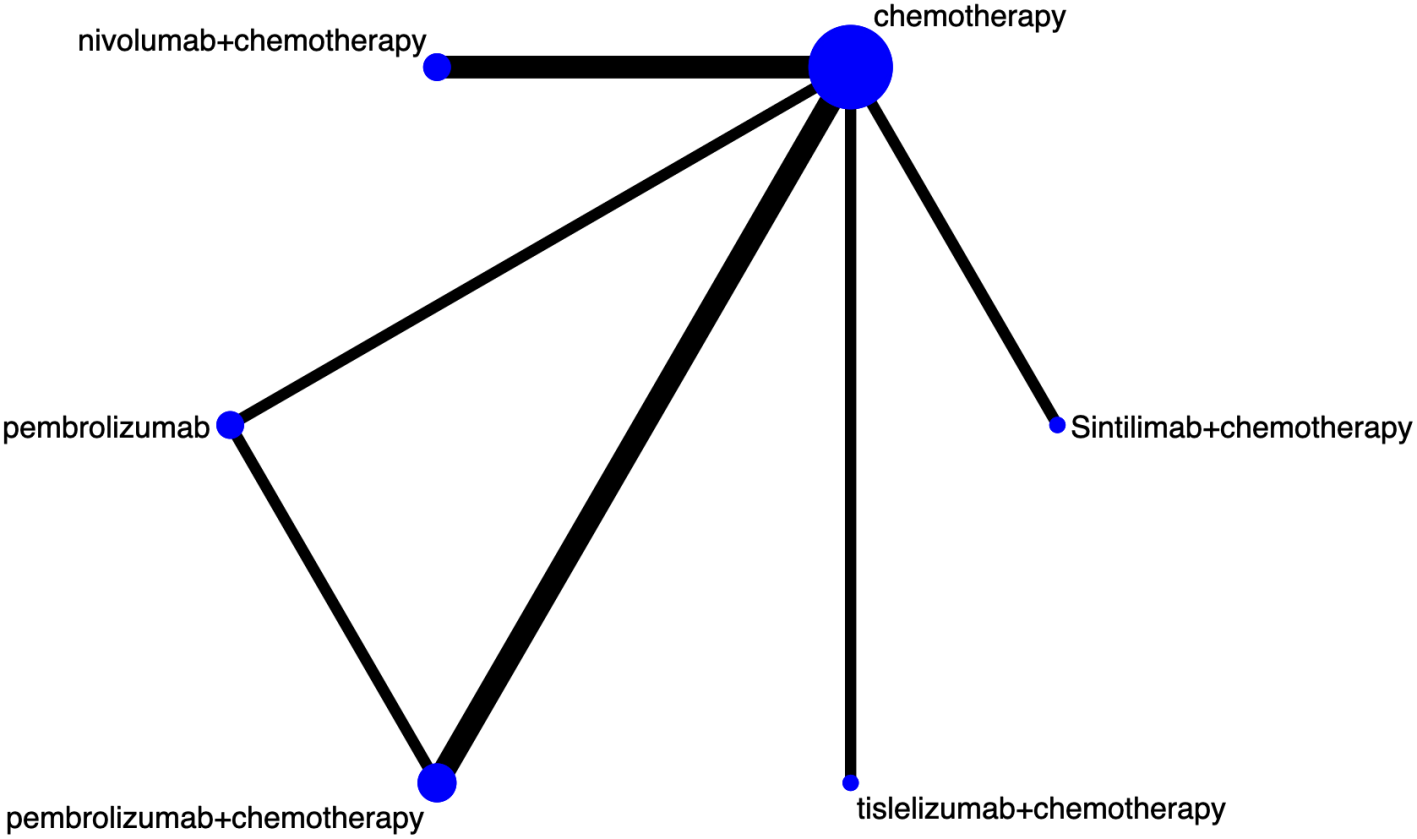
Network plots of direct comparisons for efficacy outcomes. Each node represents a treatment regimen. The size of each node is proportional to the number of patients included in the respective studies. The width of the connecting edges represents the number of RCTs.

The study selection process is outlined in **Figure 1**. Following a systematic literature search across multiple databases, removal of duplicates, and comprehensive full-text review, a total of six studies were ultimately included (4, 6, 9–12), encompassing 6294 previously untreated patients with advanced GC/GEJC. **Figure 2** illustrates the network of available direct comparisons for efficacy outcomes.

### Characteristics of the included studies

The characteristics of the included studies are comprehensively summarized in **Table 1**. All six studies were phase III RCTs. Notably, one study restricted PD-L1 expression levels, exclusively enrolling patients with PD-L1 Combined Positive Score (CPS) of ≥ 1 (6). The remaining studies included patients regardless of their PD-L1 levels. Among the 6,294 patients, 256 patients received PD-1 inhibitor monotherapy (pembrolizumab), 3,029 patients received a PD-1 inhibitor plus chemotherapy (1,047 with pembrolizumab, 1,154 with nivolumab, 327 with sintilimab, and 501 with tislelizumab), and 3,009 received either chemotherapy or chemotherapy plus placebo. The assessment of bias risk in the included studies is presented in **Supplementary Figure 1**. All studies adhered to the principle of randomization. Six studies employed low-risk randomization methods, four studies implemented allocation concealment (4, 6, 9, 10), and two studies did not describe their methods for allocation concealment (11, 12).

**Table 1:** Characteristics of included studies.

Author	Year	Trial name	Race/ethnicity	Treatment stage	Populations	Sample size	Intervention arm	Control arm	Outcomes
Kohei Shitara, et al. (6)	2020	KEYNOTE-062	Asian (24%) White (58%)	Phase III	PD-L1 CPS≥1 patients	763 (257/256/250)	Pembrolizumab 200mg +chemotherapy (FP)Q3W	Cohort 1: Pembrolizumab 200mg Q3W Cohort 2: Chemotherapy (FP) Q3W	OS, ORR, PFS, TRAEs, irAEs
Yoon-Koo Kang, et al. (9)	2022	ATTRACTION-4	Asian (100%)	Phase III	All randomized patients	724 (362/362)	Nivolumab 360mg +chemotherapy(SOX or CAPOX) Q3W	Placebo+chemotherapy (SOX or CAPOX)Q3W	OS, ORR, PFS, TRAEs
Sun Young Rha et al. (10)	2023	KEYNOTE-859	Asian (34%) White (54%)	Phase III	All randomized patients	1579 (790/789)	Pembrolizumab 200 mg +chemotherapy (CAPOX or FP) Q3W	Placebo+chemotherapy (CAPOX or FP) Q3W	OS, ORR, PFS, TRAEs, irAEs
Jianming Xu et al. (11)	2023	ORIENT-16	Asian (100%)	Phase III	All randomized patients	650 (327/323)	Sintilimab (3mg/kg for body weight <60 kg, 200mg for ≥60 kg+chemotherapy (CAPOX) Q3W	Placebo+chemotherapy (CAPOX) Q3W	OS, PFS, ORR, TRAEs, irAEs
Yelena Y Janjigian, et al. (4)	2024	CheckMate 649	Asian (25%) White (14%)	Phase III	All randomized patients	1581 (792/789)	Nivolumab 360 mg +CAPOX Q3W or 240 mg +FOLFOX Q2W	Chemotherapy (CAPOX Q3W or FOLFOX Q2W)	OS, PFS, ORR, TRAEs
Miao-Zhen Qiu, et al. (12)	2024	RATIONALE-305	Asian (75%) White (23%)	Phase III	All randomized patients	997 (501/496)	Tislelizumab 200 mg +chemotherapy (CAPOX or FP) Q3W	Chemotherapy (CAPOX or FP) Q3W	OS, PFS, ORR, TRAEs, irAEs

CPS, combined positive score; OS, overall survival; PFS, progression-free survival; ORR, objective response rate; TRAEs, treatment related adverse events; irAEs, immune-related adverse events; FP, fluorouracil+capecitabine; SOX, oxaliplatin+ S-1; CAPOX, oxaliplatin+capecitabine; FOLFOX, 5-FU+oxaliplatin+leucovorin.

### OS comparison

**Figure 3:**
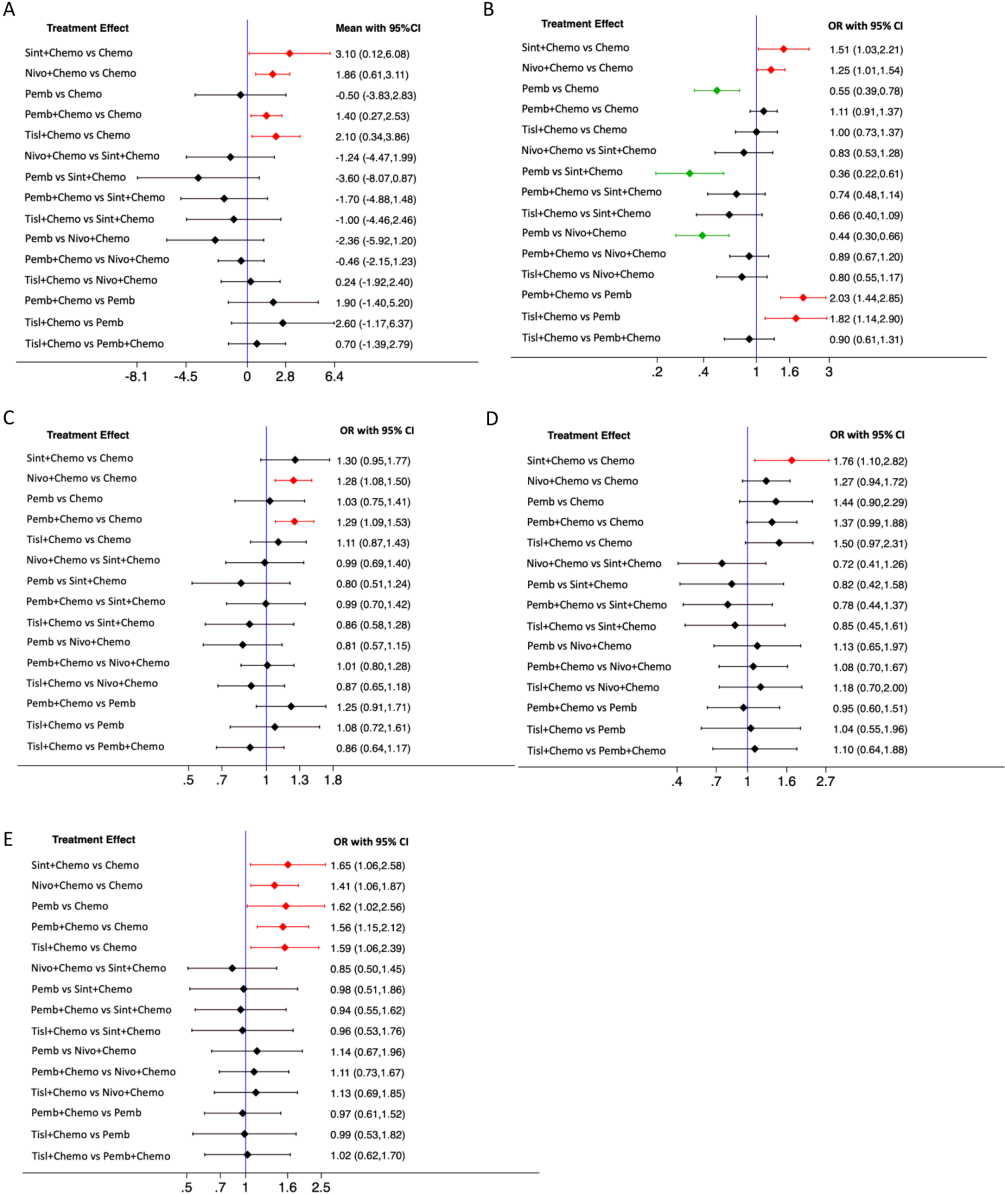
Forest plot for overall survival (OS). **(A)**, median OS; **(B)**, 6-month OS; **(C)**, 12-month OS; **(D)**, 18-month OS; **(E)**, 24-month OS.

**Table 2** presents the network rankings of OS for different treatment regimens based on the SUCRA. Sintilimab plus chemotherapy was likely to achieve the optimal OS outcomes, as evidenced by the highest SUCRA values in median OS (85.2%, MD=3.10, 95% CI 0.12-6.08), 6-month OS (92.7%, OR=1.51, 95%CI 1.03-2.21), 18-month OS (81.7%, OR=1.76, 95%CI 1.10-2.82), and 24-month OS (67.7%, OR=1.65, 95%CI 1.06-2.58) (**Supplementary Figure 2**). The forest plot for OS comparisons (**Figure 3**) indicated that compared with chemotherapy, sintilimab plus chemotherapy, pembrolizumab plus chemotherapy, nivolumab plus chemotherapy, and tislelizumab plus chemotherapy all significantly enhanced median OS outcomes. However, pembrolizumab monotherapy failed to show a significant improvement in median OS outcomes compared with chemotherapy.

**Table 2:** Network rankings of overall survival by SUCRA.

Treatment	Median OS	6-monthOS	12-month OS	18-month OS	24-month OS
Sintilimab+ Chemotherapy	**85.2**	**92.7**	72.3	**81.7**	**67.7**
Tislelizumab+ Chemotherapy	70.2	39.3	40.6	63.9	61.9
Nivolumab+ Chemotherapy	65.5	76.7	72.7	41.3	44.8
Pembrolizumab+ Chemotherapy	51	57.3	**75.3**	58.0	60.2
Pembrolizumab	14.9	0.10	25.3	51.2	64.4
Chemotherapy	13.2	33.9	13.8	3.9	1.1

OS, overall survival. The bold values mean that this treatment has the highest SUCRA value.

### PFS comparison

**Figure 4:**
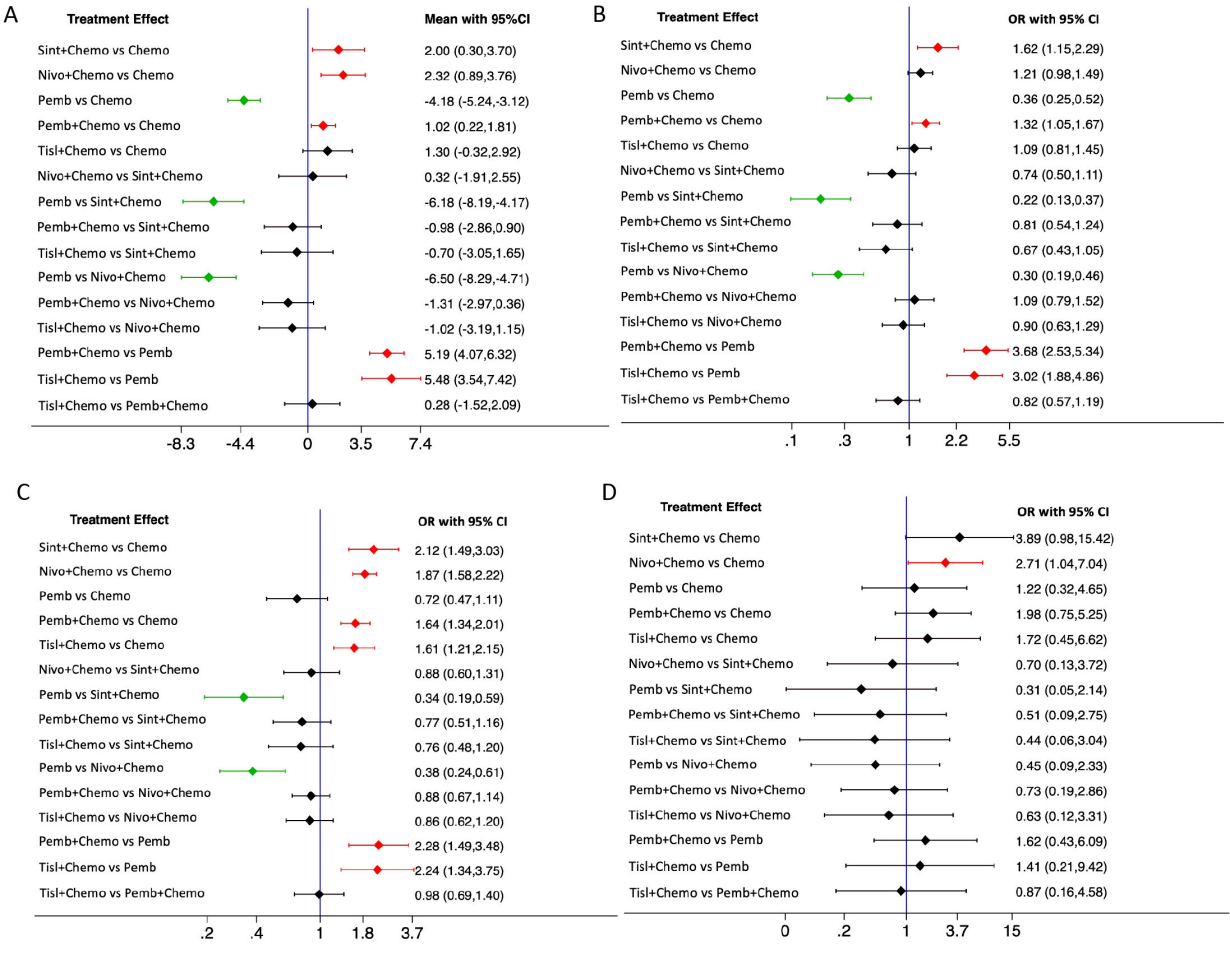
Forest plot for PFS. **(A)**, median PFS; **(B)**, 6-month PFS; **(C)**, 12-month PFS; **(D)**, 18-month PFS.

The network rankings of PFS for different treatment regimens based on the SUCRA scores are presented in **Table 3** and **Supplementary Figure 3**. For median PFS, nivolumab plus chemotherapy achieved the highest SUCRA score (96.8%, MD=2.32, 95% CI 0.89-3.76). Whereas, for 6-month PFS (94.2%, OR=1.62, 95%CI 1.15-2.29), 12-month PFS (89.9%, OR=2.12, 95%CI 1.49-3.03), and 18-month PFS (82.1%, OR=3.89, 95%CI 0.98-15.42), sintilimab plus chemotherapy exhibited the highest SUCRA scores. The forest plot for PFS comparisons (**Figure 4**) indicated that compared with chemotherapy, sintilimab plus chemotherapy, pembrolizumab plus chemotherapy, and nivolumab plus chemotherapy all significantly improved median PFS. Furthermore, the median PFS of sintilimab plus chemotherapy, pembrolizumab plus chemotherapy, nivolumab plus chemotherapy, and chemotherapy was significantly superior to that of pembrolizumab monotherapy.

**Table 3:** Network rankings of progression-free survival by SUCRA.

Treatment	Median PFS	6-month PFS	12-month PFS	18-month PFS
Nivolumab+ Chemotherapy	**96.8**	60.7	78.6	70.7
Sintilimab+ Chemotherapy	71.1	**94.2**	**89.9**	**82.1**
Pembrolizumab+ Chemotherapy	61.4	74.2	56.4	55.4
Tislelizumab+ Chemotherapy	47.8	44.0	55.1	46.8
Chemotherapy	22.9	26.9	18.5	14.4
Pembrolizumab	0	0	1.5	30.6

PFS, progression-free survival. The bold values mean that this treatment has the highest SUCRA value.

### ORR comparison

**Figure 5:**
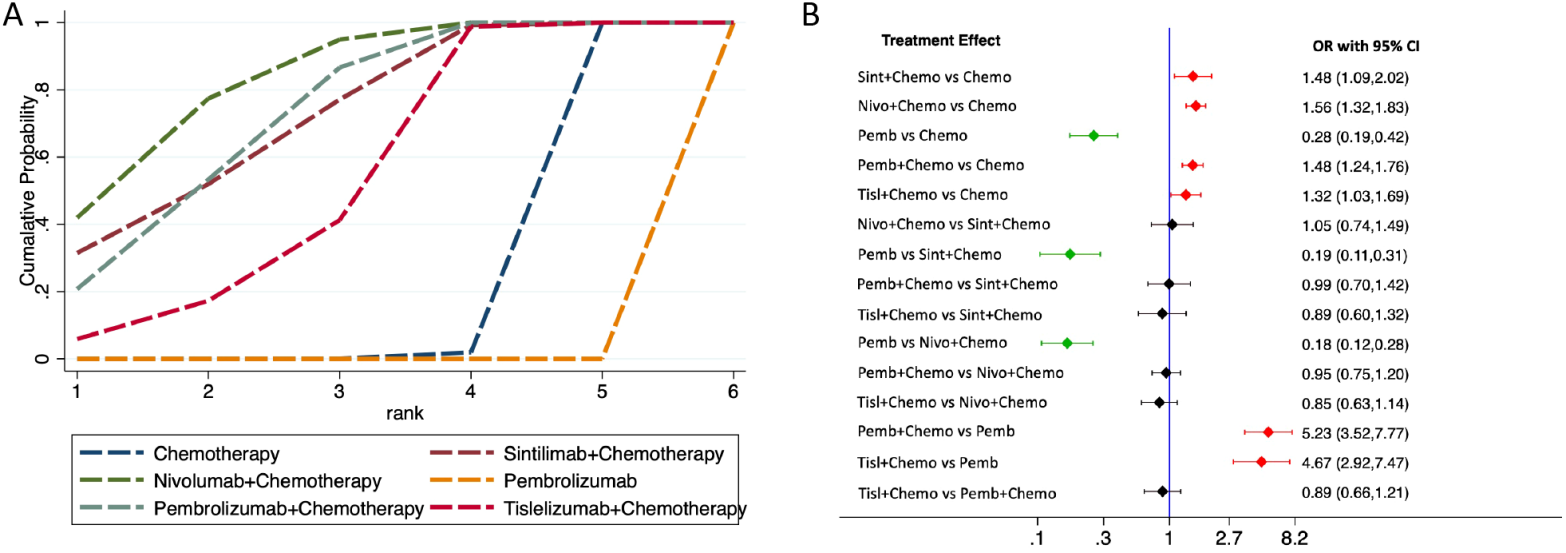
ORR comparison. **(A)**, network rankings of ORR by SUCRA; **(B)**, forest plot for ORR.

The network rankings of ORR for different treatment regimens based on the SUCRA scores are presented in **Table 4**. Nivolumab plus chemotherapy achieved the highest SUCRA value for ORR (82.9%, OR=1.56, 95%CI 1.32-1.83). The forest plot (**Figure 5**) presented that sintilimab plus chemotherapy, pembrolizumab plus chemotherapy, nivolumab plus chemotherapy, and tislelizumab plus chemotherapy all exhibited significantly higher ORRs compared with chemotherapy and pembrolizumab monotherapy.

**Table 4:** Network rankings of ORR by SUCRA.

Treatment	ORR
Nivolumab+chemotherapy	**82.9**
Pembrolizumab+chemotherapy	72.1
Sintilimab+chemotherapy	72
Tislelizumab+chemotherapy	52.6
Chemotherapy	20.4
Pembrolizumab	0

ORR, objective response rate. The bold values mean that this treatment has the highest SUCRA value.

### Subgroup analysis based on PD-L1 CPS levels

Subgroup analyses were conducted based on levels of PD-L1 CPS to compare differences in OS and PFS outcomes. Among patients with PD-L1 CPS ≥ 1, sintilimab plus chemotherapy ranked highest SUCRA value for median OS (88.7%, MD=5.80, 95% CI 1.35-10.25), while nivolumab plus chemotherapy exhibited the highest SUCRA value for median PFS (87.9%, MD=2.32, 95% CI 0.89-3.76). In patients with PD-L1 CPS ≥ 5, pembrolizumab monotherapy achieved the highest SUCRA value for median OS (81.6%, MD=7.33, 95% CI -0.01-14.67), and pembrolizumab plus chemotherapy had the highest SUCRA value for median PFS (84.9%, MD=2.50, 95% CI 1.49-3.51) (**Supplementary Figures 4A, C, E, G**). The forest plots depicting treatment comparisons (**Supplementary Figures 4B, D, F, H**) revealed that in both PD-L1 CPS ≥ 1 and PD-L1 CPS ≥ 5 patient populations, sintilimab plus chemotherapy, pembrolizumab plus chemotherapy, nivolumab plus chemotherapy, and tislelizumab plus chemotherapy significantly improved median OS compared with chemotherapy. However, among patients with PD-L1 CPS ≥ 5, no significant difference in median OS was observed between pembrolizumab monotherapy and other PD-1 inhibitor plus chemotherapy.

### Safety

**Figure 6:**
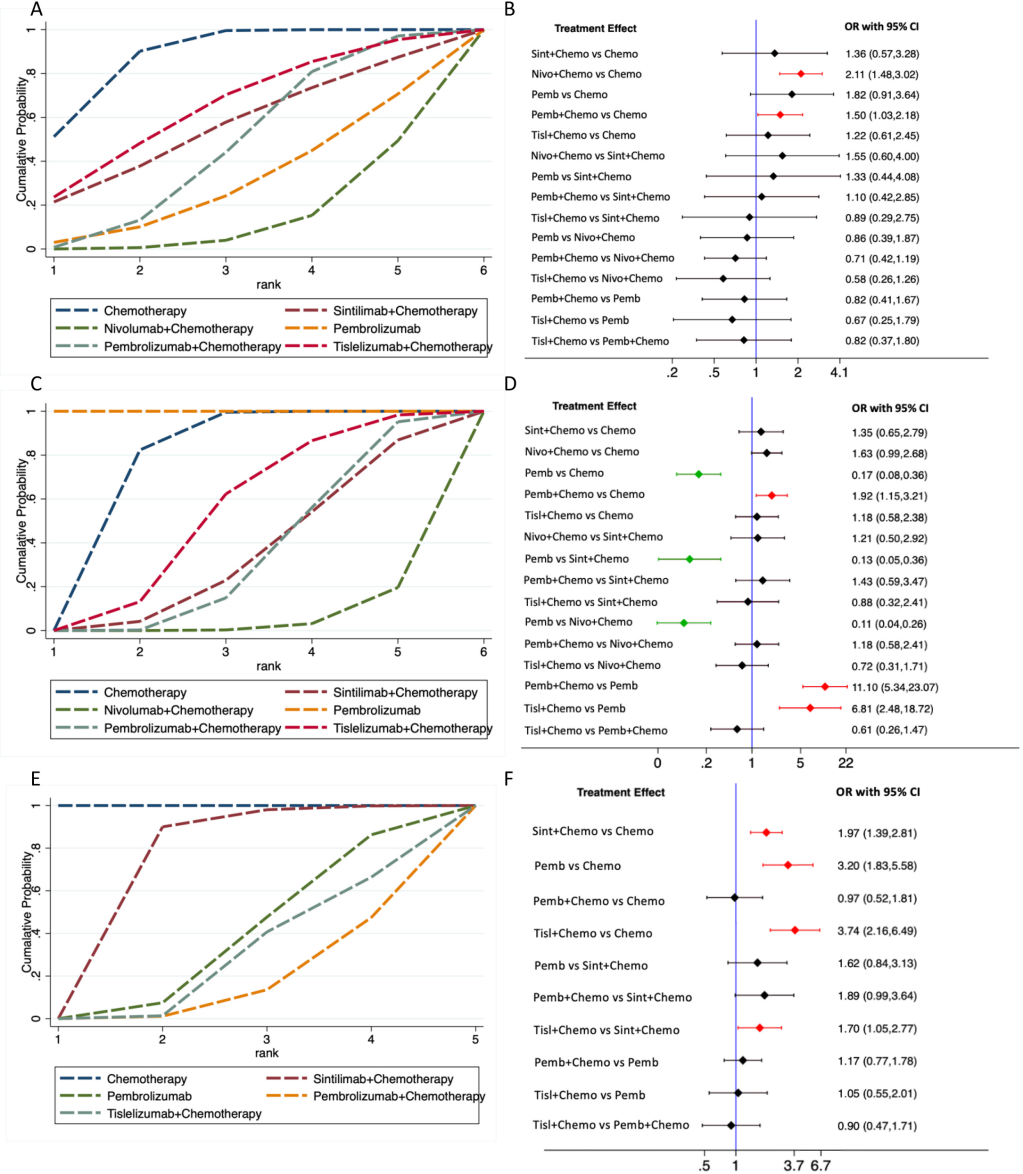
Treatment-related adverse events (TRAEs) comparison. **(A)**, network rankings of any-grade TRAEs by SUCRA; **(B)**, forest plot for any-grade TRAEs; **(C)**, network rankings of grade 3 or worse TRAEs by SUCRA; **(D)**, forest plot for grade 3 or worse TRAEs. **(E)**, network rankings of any-grade irAEs by SUCRA; **(F)**, forest plot for any-grade irAEs.

The safety analysis revealed that chemotherapy exhibited the highest SUCRA value for any-grade TRAEs, indicating the superior safety (88.3%) (**Table 5**; **Figure 6A**). The forest plot (**Figure 6B**) showed that the incidence of any-grade TRAEs was significantly elevated for both pembrolizumab plus chemotherapy and nivolumab plus chemotherapy compared with chemotherapy, whereas sintilimab plus chemotherapy, tislelizumab plus chemotherapy, and pembrolizumab monotherapy had comparable incidence rates to chemotherapy. For TRAEs of grade 3 or worse, pembrolizumab monotherapy had the highest SUCRA value (100%, OR=0.17, 95%CI 0.08-0.36), signifying the best safety (**Figure 6C**). The forest plot (**Figure 6D**) revealed that the rates of TRAEs of grade 3 or worse were significantly lower for pembrolizumab monotherapy compared with sintilimab plus chemotherapy, pembrolizumab plus chemotherapy, nivolumab plus chemotherapy, tislelizumab plus chemotherapy, and chemotherapy. Conversely, the rate of grade 3 or worse events for pembrolizumab plus chemotherapy was significantly higher than that for chemotherapy.The other three PD-1 inhibitors plus chemotherapy did not increase grade 3 or worse TRAEs compared with chemotherapy.

**Table 5:** Network rankings of safety outcomes by SUCRA.

Treatment	Any-grade treatment-related adverse events	Grade 3 or worse treatment-related adverse events	Any-grade immune-related adverse events
Chemotherapy	**88.3**	76.4	**100**
Tislelizumab+Chemotherapy	65.1	52.1	27.1
Sintilimab+Chemotherapy	55	33.6	72.0
Pembrolizumab+Chemotherapy	47.2	33.3	15.6
Pembrolizumab	30.3	**100**	35.4
Nivolumab+Chemotherapy	14	4.6	–

The bold values mean that this treatment has the highest SUCRA value.

Chemotherapy exhibited the highest SUCRA value for irAEs, followed by sintilimab plus chemotherapy, which had an SUCRA value of 72.0% (OR=1.97, 95%CI 1.39-2.81), the highest among all PD-1 inhibitor plus chemotherapy treatments (**Figure 6E**). This suggested that sintilimab had the best safety profile against irAEs. Forest plot results indicated that sintilimab plus chemotherapy, pembrolizumab monotherapy and tislelizumab plus chemotherapy all increased the risk of irAEs compared to chemotherapy. Furthermore, tislelizumab plus chemotherapy increased the risk of irAEs compared with sintilimab plus chemotherapy (**Figure 6F**).

## Discussions

The results of this network meta-analysis suggested that, in terms of efficacy, PD-1 inhibitor plus chemotherapy significantly improved the OS, PFS, and ORR in patients with advanced GC/GEJC, when compared with chemotherapy as a first-line treatment. However, pembrolizumab monotherapy did not improve OS compared with chemotherapy, and its median PFS was significantly lower than in the chemotherapy group. Moreover, network rankings analysis of different PD-1 inhibitors revealed that sintilimab plus chemotherapy had the highest SUCRA value for OS (85.2%, MD=3.10, 95% CI 0.12-6.08), while nivolumab plus chemotherapy had the highest SUCRA values for both PFS (96.8%, MD=2.32, 95% CI 0.89-3.76) and ORR (82.9%, OR=1.56, 95%CI 1.32-1.83). In terms of safety, compared with chemotherapy, pembrolizumab monotherapy significantly reduced the incidence of grade 3 or worse TRAEs, indicating superior safety over chemotherapy. Similarly, tislelizumab plus chemotherapy, as well as sintilimab plus chemotherapy, did not increase the overall incidence and the incidence of grade 3 or worse TRAEs, suggesting good safety. For nivolumab plus chemotherapy, while the overall incidence of TRAEs increased, there was no significant rise in the incidence of grade 3 or worse TRAEs. Conversely, pembrolizumab plus chemotherapy led to an increase in both the overall incidence and the incidence of grade 3 or worse TRAEs. While SUCRA provides a ranking framework, it does not reflect the magnitude of treatment effects. Interpretation should consider both SUCRA values and effect sizes, particularly in cases where small differences exist between treatments. Our results show that SUCRA values are consistent with the effect sizes. However, our findings should be interpreted with caution, as limited direct evidence was highly represented among the included studies.

To our knowledge, there are no studies that comprehensively compare the safety and efficacy of different PD-1 inhibitors for advanced GC/GEJC. Previous studies have compared the efficacy of PD-1 inhibitors plus chemotherapy versus chemotherapy alone (13–15). For example, Zhang et al. (14) included nine phase 3 clinical trials and found that PD-1 inhibitor plus chemotherapy significantly prolonged OS compared with chemotherapy alone (hazard ratio [HR], 0.76; 95% CI, 0.71-0.81). Another meta-analysis also reported that immune checkpoint inhibitors (ICIs) plus chemotherapy improved OS (HR, 0.86; 95% CI 0.78-0.94), PFS (HR, 0.79; 95% CI 0.63-0.99) and ORR (relative ratio [RR], 1.20; 95% CI 1.11-1.30) (15). A real-world study from 13 medical institutions also showed significant improvements in PFS and OS with ICIs plus chemotherapy in advanced GC (16). These results are similar with our findings that PD-1 inhibiors plus chemotherapy improve OS and PFS in advanced GC/GEJC. Futhermore, we conducted a network meta-analysis to compare pairwise differences between various treatment regimens. SUCRA values were utilized to rank the efficacy and safety of these treatment regimens. Our results demonstrated that sintilimab plus chemotherapy had the highest SUCRA value for OS at 85.2%, whereas nivolumab plus chemotherapy had the highest SUCRA values for both PFS and ORR at 96.8% and 82.9%, respectively. These findings suggest the therapeutic advantages of sintilimab and nivolumab.

This study indicates that the administration of PD-1 inhibitor pembrolizumab as monotherapy leads to a significantly lower ORR when compared with chemotherapy and PD-1 inhibitor plus chemotherapy. This finding suggests that PD-1 inhibitor monotherapy may not enhance ORR and necessitates integration with chemotherapy. Current Phase III studies indicate that the ORR for first-line chemotherapy in patients with advanced GC/GEJC has seemingly reached a ceiling, with an ORR that is unlikely to exceed 40% to 50%. In contrast, PD-1 inhibitor plus chemotherapy has achieved an ORR ranging from 47.1% to 85%, significantly improving the ORR among patients with advanced GC/GEJC (17).

The expression level of PD-L1 is widely recognized as a crucial biomarker for predicting the efficacy of PD-1/PD-L1 inhibitors. Kim et al. (18) demonstrated that patients with PD-L1-positive tumors exhibited a significantly higher ORR (50% vs. 0%, P < 0.001) than those with PD-L1-negative tumors. In the RATIONALE-305 trial (12), tislelizumab plus chemotherapy resulted in longer median OS and median PFS in patients with a PD-L1 TAP score ≥5%, compared with overall randomized patient population (17.2 months [13.9 to 21.3] vs. 15.0 months [13.6 to 16.5]; 7.2 months [5.8 to 8.4] vs. 6.9 months [5.7 to 7.2], respectively). Furthermore, the ORIENT-16 trial (11) confirmed that sintilimab plus chemotherapy achieved longer median OS and median PFS in patients with a PD-L1 CPS ≥5, compared with all randomized patients (18.4 months vs. 15.2 months; 7.8 months vs. 7.1 months, respectively). Our network meta-analysis, based on subgroup analyses stratified by PD-L1 CPS levels, revealed that the ranking of efficacy and safety varied among different PD-1 inhibitors in patients with different PD-L1 CPS levels. Overall, regardless of the patient’s PD-L1 CPS levels, the addition of PD-1 inhibitors to chemotherapy significantly improved OS and PFS in untreated patients with advanced GC/GEJC.

Treatment with PD-1 inhibitors in combination with chemotherapy generally exhibits a considerable safety when compared with chemotherapy. Compared to chemotherapy, nivolumab, sintilimab, and tislelizumab combined with chemotherapy did not increase the incidence of grade 3 or worse of TRAEs. Furthermore, sintilimab and tislelizumab did not increase the incidence of any grade TRAEs. These observations suggest that sintilimab and tislelizumab can be considered to have a better safety compared with pembrolizumab and nivolumab. Additionally, irAEs are a significant consideration during treatment with PD-1 inhibitors. Our findings indicated variations in irAEs among different PD-1 inhibitors. Specifically, compared to sintilimab plus chemotherapy, tislelizumab plus chemotherapy increased the risk of irAEs. However, caution is warranted in interpreting these safety outcomes. The distinction between TRAEs and irAEs may arise from baseline differences among the study populations. A study utilizing the US Food and Drug Administration Adverse Event Reporting System (FAERS) database demonstrated an increased level of toxicities in older patients than in their younger counterparts when treated with anti-PD-(L)1 agents (19). In the studies we reviewed, the median age of patients was 60 years in the RATIONALE-305 (12), 62 years in ORIENT-16 (11), and 64 years in ATTRACTION-4 (9). Differences in patient age, and the proportion of patients older than 65 years may contribute to variations inTRAEs, particularly irAEs, due to age-related alterations in immune cell function (20). Furthermore, irAEs may also be associated with body mass index (21), ethnic differences (22), and treatment parameters. For example, in the CheckMate 649 trial, nivolumab was administered biweekly in combination with FOLFOX (240 mg every 2 weeks), potentially leading to an increased incidence of infusion-associated irAEs compared to the triweekly regimen utilized in other studies.

This network meta-analysis revealed that the safety of pembrolizumab monotherapy was superior to chemotherapy, with a reduced incidence of grade 3 or worse TRAEs (OR, 0.17; 95%CI, 0.08, 0.36). Notably, our analysis did not include studies that investigated other PD-1 inhibitor monotherapy as an intervention. Similarly, in the JAVELIN Gastric 300 study (23), avelumab demonstrated a significantly lower rate of grade 3 or worse TRAEs compared with chemotherapy in the third-line treatment of GC (9.2% vs. 31.6%). These results support the consideration of PD-1 inhibitor monotherapy as a treatment option for untreated patients with advanced GC/GEJC who were unsiutable for chemotherapy.

We observed notable variations in the median OS among patients receiving the same treatment regimen, such as chemotherapy, across different studies. For instance, in the KEYNOTE-062 trial (6), the median OS for patients in the chemotherapy group was 11.1 months, whereas in the ATTRACTION-4 trial (9), the median OS for patients treated with chemotherapy was 17.15 months. These differences may be attributed to regional variations in medical conditions and practices, as well as differences in patient populations and treatment adherence. Notably, the ATTRACTION-4 trial exclusively enrolled Asian patients, while the proportion of Asian patients in the KEYNOTE-062 trial was approximately 24%. Asian patients generally receive more subsequent anticancer therapies, which may contribute to their relatively better survival prognosis (9, 24–26).

The primary strength of this study is its comprehensive comparison of the efficacy and safety of six treatment regimens, including four PD-1 inhibitor monotherapy and their combinations with chemotherapy. Furthermore, all included studies were large-scale RCTs with high methodological quality, contributing to a robust level of evidence. However, it is essential to acknowledge the following limitations: (1) As previously discussed, the geographical distribution of trials and the inherent differences in medical practices across regions may potentially influence OS and PFS outcomes. Unfortunately, there was a lack of subgroup analysis data separating Asian and non-Asian populations in the included studies, which limited the exploration of the influence of population factors on the effectiveness and safety of different PD-1 inhibitors. (2) Differences in the dose and frequency of PD-1 inhibitors, as well as the combination of chemotherapy agents, hinder subgroup analyses based on specific drug regimens. Additionally, the concurrent use of chemotherapy drugs may introduce bias into the evaluation of efficacy and safety outcomes. Variations in chemotherapy protocols, such as the choice of drugs, dosages, and schedules, could contribute to heterogeneity in the treatment effects across trials. These factors should be considered when interpreting the findings of our study, and further investigation is warranted to evaluate whether different concomitant chemotherapy regiments will affect the outcome of PD-1 inhibitors. (3) Given that only six RCTs included, the overall sample size remains modest, highlighting the need for additional RCTs to reinforce the findings and provide a more comprehensive understanding.

## Conclusions

In the first-line treatment of advanced GC/GEJC, the combination of PD-1 inhibitors with chemotherapy has been demonstrated to significantly improve OS, PFS, and ORR compared with chemotherapy. Among them, sintilimab plus chemotherapy achieved the highest SUCRA value for OS, and nivolumab plus chemotherapy achieved the highest SUCRA value for PFS and ORR. Regarding safety, tislelizumab plus chemotherapy and sintilimab plus chemotherapy did not increase the overall incidence and the incidence of grade 3 or worse TRAEs, demonstrating good tolerability and safety.

## Supplementary material

Supplementary Figure 1 Risk-of-bias assessments for included studies.

Supplementary Figure 2 Network rankings of overall survival by SUCRA. **(A)**, median OS; **(B)**,6-month OS; **(C)**, 12-month OS; **(D)**, 18-month OS; **(E)**, 24-month OS.

Supplementary Figure 3 Network rankings of PFS by SUCRA. **(A)**, median PFS; **(B)**, 6-month PFS; **(C)**, 12-month PFS; **(D)**, 18-month PFS.

Supplementary Figure 4 OS and PFS comparison stratified by PD-L1 CPS. **(A)**, network rankings of OS by SUCRA in patients with PD-L1 CPS ≥1; **(B)**, forest plot for OS in patients with PD-L1 CPS ≥1; **(C)**, network rankings of PFS by SUCRA in patients with PD-L1 CPS ≥1; **(D)**, forest plot for PFS in patients with PD-L1 CPS ≥1; **(E)**, network rankings of OS by SUCRA in patients with PD-L1 CPS ≥5; **(F)**, forest plot for OS in patients with PD-L1 CPS ≥5; **(G)**, network rankings of PFS by SUCRA in patients with PD-L1 CPS ≥5; **(H)**, forest plot for PFS in patients with PD-L1 CPS ≥5.

## References

[1] Sung HA-O Ferlay J Siegel RA-O Laversanne M Soerjomataram I Jemal A. Global cancer statistics 2020: GLOBOCAN estimates of incidence and mortality worldwide for 36 cancers in 185 countries. CA Cancer J Clin. (2021) 71:209–49. doi: 10.3322/caac.21660 33538338

[2] Cao W Chen HD Yu YW Li N Chen WQ. Changing profiles of cancer burden worldwide and in China: a secondary analysis of the global cancer statistics 2020. Chin Med J (Engl). (2021) 134:783–91. doi: 10.1097/cm9.0000000000001474 PMC8104205 33734139

[3] Smyth EC Nilsson M Grabsch HI van Grieken NC Lordick F. Gastric cancer. Lancet. (2020) 396:635–48. doi: 10.1016/s0140-6736(20)31288-5 32861308

[4] Janjigian YY Ajani JA Moehler M Shen L Garrido M Gallardo C. First-line nivolumab plus chemotherapy for advanced gastric, gastroesophageal junction, and esophageal adenocarcinoma: 3-year follow-up of the phase III checkmate 649 trial. J Clin Oncol. (2024) 42:2012–20. doi: 10.1200/jco.23.01601 PMC11185916 38382001

[5] Pitt JM Vétizou M Daillère R Roberti MP Yamazaki T Routy B. Resistance mechanisms to immune-checkpoint blockade in cancer: tumor-intrinsic and -extrinsic factors. Immunity. (2016) 44:1255–69. doi: 10.1016/j.immuni.2016.06.001 27332730

[6] Shitara K Van Cutsem E Bang YJ Fuchs C Wyrwicz L Lee KW. Efficacy and safety of pembrolizumab or pembrolizumab plus chemotherapy vs chemotherapy alone for patients with first-line, advanced gastric cancer: the KEYNOTE-062 phase 3 randomized clinical trial. JAMA Oncol. (2020) 6:1571–80. doi: 10.1001/jamaoncol.2020.3370 PMC7489405 32880601

[7] Hegewisch-Becker SA-O Mendez G Chao JA-OX Nemecek R Feeney K Van Cutsem EA-O. First-line nivolumab and relatlimab plus chemotherapy for gastric or gastroesophageal junction adenocarcinoma: the phase II RELATIVITY-060 study. J Clin Oncol. (2024) 42:2080–93. doi: 10.1200/jco.23.01636 PMC11191068 38723227

[8] Salanti G Ades-Ae-Fau-Ioannidis JPA Ioannidis JP. Graphical methods and numerical summaries for presenting results from multiple-treatment meta-analysis: an overview and tutorial. J Clin Epidemiol. (2011) 64:163–71. doi: 10.1016/j.jclinepi.2010.03.016 20688472

[9] Kang YK Chen LT Ryu MH Oh DY Oh SC Chung HC. Nivolumab plus chemotherapy versus placebo plus chemotherapy in patients with HER2-negative, untreated, unresectable advanced or recurrent gastric or gastro-oesophageal junction cancer (ATTRACTION-4): a randomised, multicentre, double-blind, placebo-controlled, phase 3 trial. Lancet Oncol. (2022) 23:234–47. doi: 10.1016/s1470-2045(21)00692-6 35030335

[10] Rha SY Oh DY Yañez P Bai Y Ryu MH Lee J. Pembrolizumab plus chemotherapy versus placebo plus chemotherapy for HER2-negative advanced gastric cancer (KEYNOTE-859): a multicentre, randomised, double-blind, phase 3 trial. Lancet Oncol. (2023) 24:1181–95. doi: 10.1016/s1470-2045(23)00515-6 37875143

[11] Xu J Jiang H Pan Y Gu K Cang S Han L. Sintilimab plus chemotherapy for unresectable gastric or gastroesophageal junction cancer: the ORIENT-16 randomized clinical trial. Jama. (2023) 330:2064–74. doi: 10.1001/jama.2023.19918 PMC10698618 38051328

[12] Qiu MZ Oh DY Kato K Arkenau T Tabernero J Correa MC. Tislelizumab plus chemotherapy versus placebo plus chemotherapy as first line treatment for advanced gastric or gastro-oesophageal junction adenocarcinoma: RATIONALE-305 randomised, double blind, phase 3 trial. Bmj. (2024) 385:e078876. doi: 10.1136/bmj-2023-078876 38806195

[13] Noori M Fayyaz F Zali MR Bashash DA-O. Predictive value of PD-L1 expression in response to immune checkpoint inhibitors for gastric cancer treatment: a systematic review and meta-analysis. Expert Rev Anticancer Ther. (2023) 23:1029–39. doi: 10.1080/14737140.2023.2238896 37466449

[14] Zhang XM Yang T Xu YY Li BZ Shen W Hu WQ. Effectiveness and tolerability of programmed cell death protein-1 inhibitor + chemotherapy compared to chemotherapy for upper gastrointestinal tract cancers. World J Gastrointest Oncol. (2024) 16:1613–25. doi: 10.4251/wjgo.v16.i4.1613 PMC11037061 38660631

[15] Zhang L Huang L Liu Z Ling TA-O. Immune checkpoint inhibitor plus chemotherapy as first-line treatment for advanced gastric or gastroesophageal junction cancer: A systematic review and meta-analysis. Technol Cancer Res Treat. (2024) 23:15330338241273286. doi: 10.1177/15330338241273286 39110075 PMC11307348

[16] Zhang X Dai X Liu A Sun M Cong L Liang J. Efficacy, safety, and biomarker analysis of first-line immune checkpoint inhibitors with chemotherapy versus chemotherapy for advanced gastric cancer: a multicenter, retrospective cohort study. BMC Med. (2024) 22:585. doi: 10.1186/s12916-024-03801-5 39696266 PMC11657984

[17] Guo X Yang B He L Sun Y Song Y Qu X. PD-1 inhibitors plus oxaliplatin or cisplatin-based chemotherapy in first-line treatments for advanced gastric cancer: A network meta-analysis. Front Immunol. (2022) 13:905651. doi: 10.3389/fimmu.2022.905651 36003374 PMC9393421

[18] Kim ST Cristescu RA-O Bass AJ Kim KA-O Odegaard JI Kim KA-O. Comprehensive molecular characterization of clinical responses to PD-1 inhibition in metastatic gastric cancer. Nat Med. (2018) 24:1449–58. doi: 10.1038/s41591-018-0101-z 30013197

[19] Huang X Tian T Zhang Y Zhou S Hu P Zhang J. Age-associated changes in adverse events arising from anti-PD-(L)1 therapy. Front Oncol. (2021) 11:619385. doi: 10.3389/fonc.2021.619385 34055598 PMC8155669

[20] Mitchell WA Lang PO Aspinall R. Tracing thymic output in older individuals. Clin Exp Immunol. (2010) 161:497–503. doi: 10.1111/j.1365-2249.2010.04209.x PMC2962967 20646007

[21] Huang YA-O Soon YY Aminkeng F Tay SH Ang Y Kee ACL. Risk factors for immune-related adverse events from anti-PD-1 or anti-PD-L1 treatment in an Asian cohort of nonsmall cell lung cancer patients. Int J Cancer. (2022) 150:636–44. doi: 10.1002/ijc.33822 34562273

[22] Lee J Sun JM Lee SH Ahn JS Park K Ahn MJ. Are there any ethnic differences in the efficacy and safety of immune checkpoint inhibitors for treatment of lung cancer? J Thorac Dis. (2020) 12:3796–803. doi: 10.21037/jtd.2019.08.29 PMC7399433 32802459

[23] Bang YJ Ruiz EY Van Cutsem E Lee KW Wyrwicz L Schenker M. Phase III, randomised trial of avelumab versus physician’s choice of chemotherapy as third-line treatment of patients with advanced gastric or gastro-oesophageal junction cancer: primary analysis of JAVELIN Gastric 300. Ann Oncol. (2018) 29:2052–60. doi: 10.1093/annonc/mdy264 PMC6225815 30052729

[24] Fuchs CS Shitara K Di Bartolomeo M Lonardi S Al-Batran SE Van Cutsem E. Ramucirumab with cisplatin and fluoropyrimidine as first-line therapy in patients with metastatic gastric or junctional adenocarcinoma (RAINFALL): a double-blind, randomised, placebo-controlled, phase 3 trial. Lancet Oncol. (2019) 20:420–35. doi: 10.1016/s1470-2045(18)30791-5 30718072

[25] Hecht JR Bang YJ Qin SK Chung HC Xu JM Park JO. Lapatinib in combination with capecitabine plus oxaliplatin in human epidermal growth factor receptor 2-positive advanced or metastatic gastric, esophageal, or gastroesophageal adenocarcinoma: TRIO-013/LOGiC–A randomized phase III trial. J Clin Oncol. (2016) 34:443–51. doi: 10.1200/jco.2015.62.6598 26628478

[26] Catenacci DVT Tebbutt NC Davidenko I Murad AM Al-Batran SE Ilson DH. Rilotumumab plus epirubicin, cisplatin, and capecitabine as first-line therapy in advanced MET-positive gastric or gastro-oesophageal junction cancer (RILOMET-1): a randomised, double-blind, placebo-controlled, phase 3 trial. Lancet Oncol. (2017) 18:1467–82. doi: 10.1016/s1470-2045(17)30566-1 PMC5898242 28958504

