# Supplementary figures and images for: Efficacy and safety of programmed cell death protein-1 inhibitor for first-line therapy of advanced gastric or gastroesophageal junction cancer: a network meta-analysis
Source: Front Immunol. 2025 Apr 8;16:1500954. doi: 10.3389/fimmu.2025.1500954 (PMC12011870; doi:10.3389/fimmu.2025.1500954)

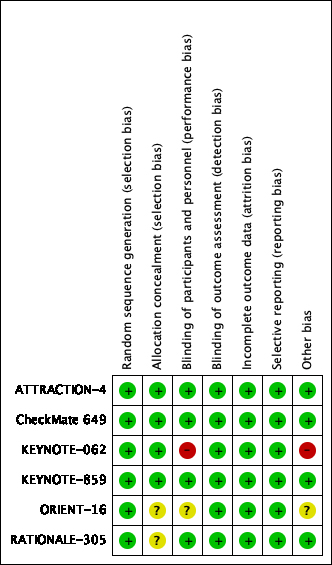

Supplement: Supplementary Figure 1 — Risk-of-bias assessments for included studies. [file Image1.jpeg]

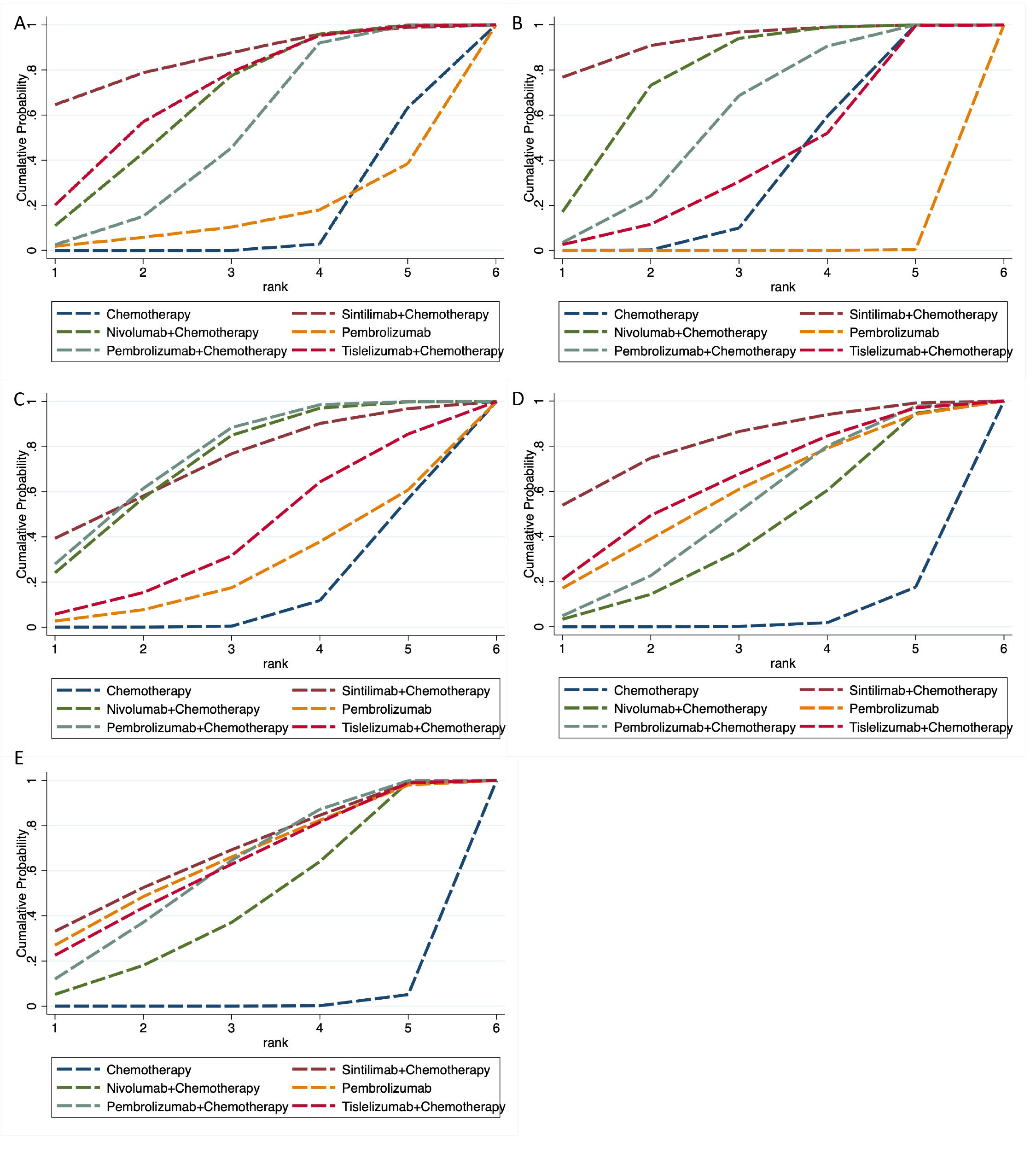

Supplement: Supplementary Figure 2 — Network rankings of overall survival by SUCRA. (A), median OS; (B),6-month OS; (C), 12-month OS; (D), 18-month OS; (E), 24-month OS. [file Image2.jpeg]

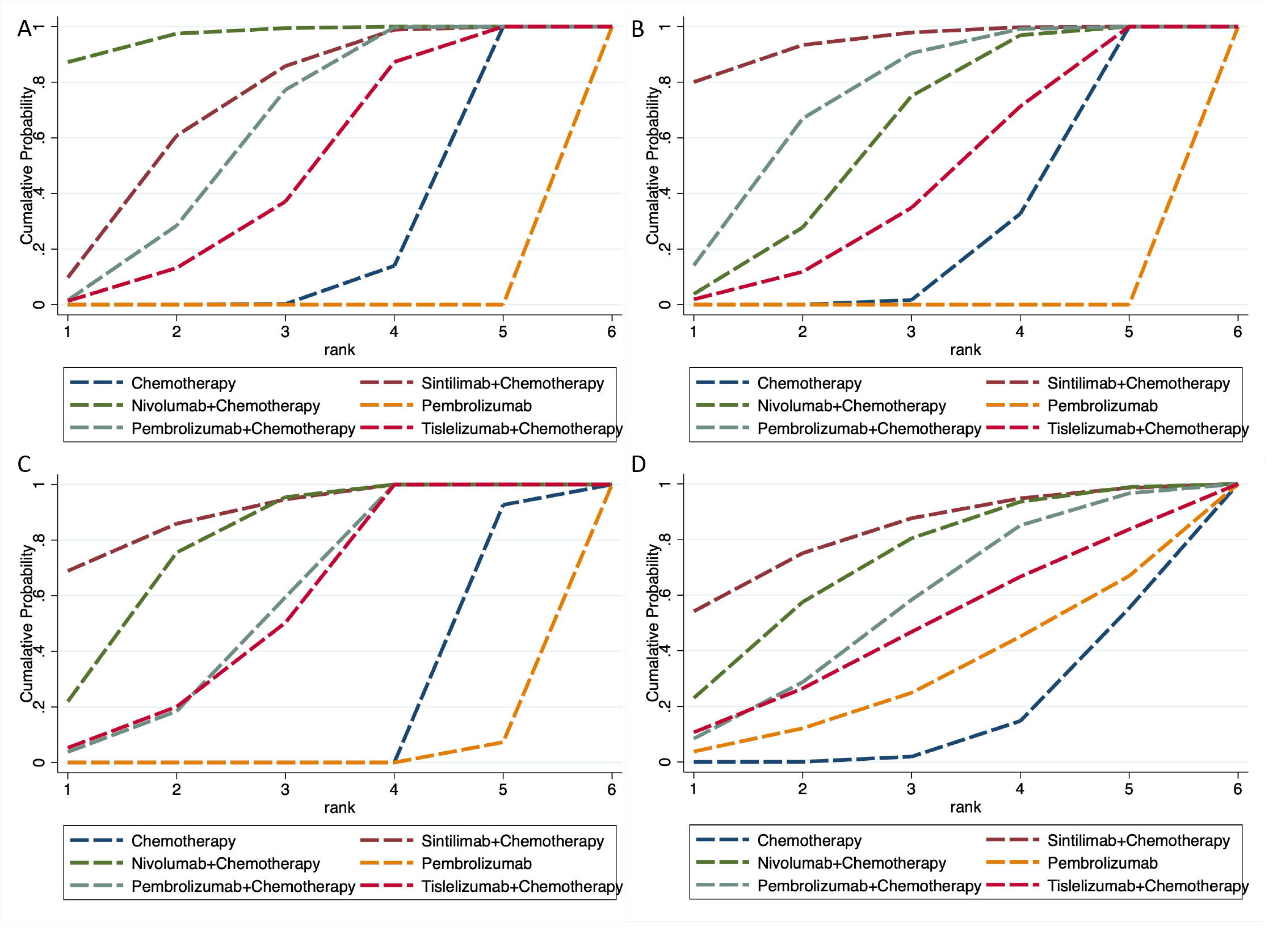

Supplement: Supplementary Figure 3 — Network rankings of PFS by SUCRA. (A), median PFS; (B), 6-month PFS; (C), 12-month PFS; (D), 18-month PFS. [file Image3.jpeg]

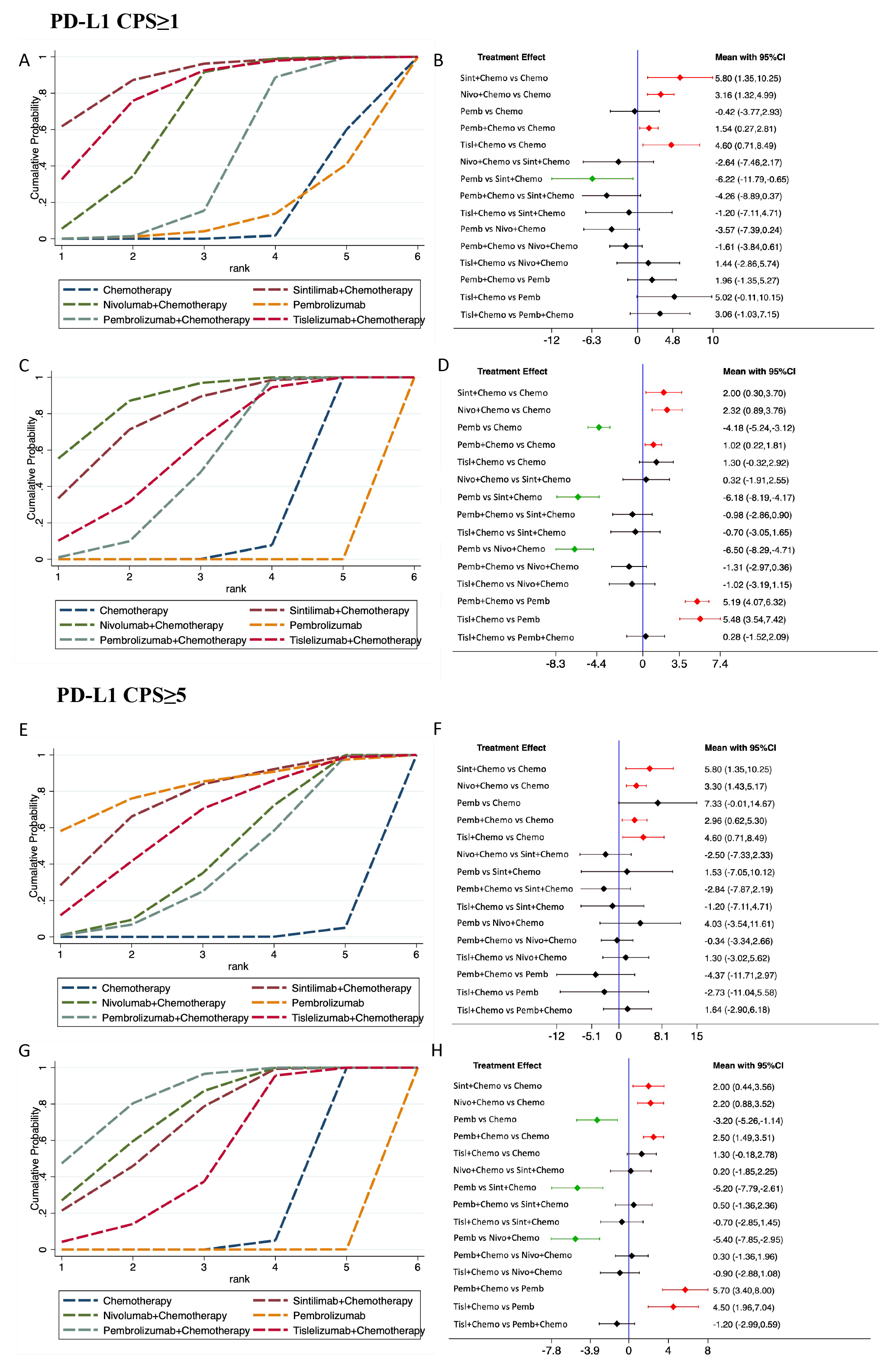

Supplement: Supplementary Figure 4 — OS and PFS comparison stratified by PD-L1 CPS. (A), network rankings of OS by SUCRA in patients with PD-L1 CPS ≥1; (B), forest plot for OS in patients with PD-L1 CPS ≥1; (C), network rankings of PFS by SUCRA in patients with PD-L1 CPS ≥1; (D), forest plot for PFS in patients with PD-L1 CPS ≥1; (E), network rankings of OS by SUCRA in patients with PD-L1 CPS ≥5; (F), forest plot for OS in patients with PD-L1 CPS ≥5; (G), network rankings of PFS by SUCRA in patients with PD-L1 CPS ≥5; (H), forest plot for PFS in patients with PD-L1 CPS ≥5. [file Image4.jpeg]
