# Supplementary material for: Efficacy and safety of programmed cell death protein-1 inhibitor for first-line therapy of advanced gastric or gastroesophageal junction cancer: a network meta-analysis
Source: Front Immunol. 2025 Apr 8;16:1500954. doi: 10.3389/fimmu.2025.1500954 (PMC12011870; doi:10.3389/fimmu.2025.1500954)
Supplement: Supplementary file 5 [file Table1.docx]

Supplementary table 1. search strategy

| Databased | Words |
| --- | --- |
| PubMed | (((((((((((((((((Neoplasm, Stomach[Title/Abstract]) OR (Stomach Neoplasm[Title/Abstract])) OR (Neoplasms, Stomach[Title/Abstract])) OR (Gastric Neoplasms[Title/Abstract])) OR (Gastric Neoplasm[Title/Abstract])) OR (Neoplasms, Gastric[Title/Abstract])) OR (Cancer of Stomach[Title/Abstract])) OR (Stomach Cancers[Title/Abstract])) OR (Gastric Cancer[Title/Abstract])) OR (Cancer, Gastric[Title/Abstract])) OR (Cancers, Gastric[Title/Abstract])) OR (Gastric Cancers[Title/Abstract])) OR (Stomach Cancer[Title/Abstract])) OR (Cancers, Stomach[Title/Abstract])) OR (Cancer of the Stomach[Title/Abstract])) OR (Cancer of the Stomach[Title/Abstract])) OR ("Stomach Neoplasms"[Mesh])) AND (randomized controlled trial[Publication Type] OR randomized[Title/Abstract] OR placebo[Title/Abstract]) |
| Cochrane Database | #1 MeSH descriptor: [Stomach Neoplasms] explode all trees  #2 MeSH descriptor: [Stomach Neoplasms] explode all trees  #3 (Neoplasm, Stomach):ti,ab,kw OR (Stomach Neoplasm):ti,ab,kw OR (Neoplasms, Stomach):ti,ab,kw OR (Gastric Neoplasms):ti,ab,kw OR (Gastric Neoplasm):ti,ab,kw (Word variations have been searched)  #4 (Neoplasm, Gastric):ti,ab,kw OR (Cancer of Stomach):ti,ab,kw OR (Stomach Cancers):ti,ab,kw OR (Gastric Cancer):ti,ab,kw OR (Cancer, Gastric):ti,ab,kw (Word variations have been searched)  #5 (Cancers, Gastric):ti,ab,kw OR (Gastric Cancers):ti,ab,kw OR (Stomach Cancer):ti,ab,kw OR (Cancer, Stomach):ti,ab,kw OR (Cancers, Stomach):ti,ab,kw (Word variations have been searched)  #6 (Cancer of the Stomach):ti,ab,kw OR (Gastric Cancer, Familial Diffuse):ti,ab,kw (Word variations have been searched)  #7 #2 or #3 or #4 or #5 or #6 |
| Embase | #1 ('stomach'/exp OR stomach) AND ('neoplasms'/exp OR neoplasms)  #2 'stomach cancer'/exp OR 'stomach cancer'  #3 'stomach cancer'/exp  #4 'neoplasm, stomach':ab,ti  #5 'stomach neoplasms':ab,ti  #6 'stomach neoplasm':ab,ti  #7 'neoplasms, stomach':ab,ti  #8 'gastric neoplasms':ab,ti  #9 'gastric neoplasm':ab,ti  #10 'neoplasm, gastric':ab,ti  #11 'neoplasms, gastric':ab,ti  #12 'cancer of stomach':ab,ti  #13 'stomach cancers':ab,ti  #14 'gastric cancer':ab,ti  #15 'cancer, gastric':ab,ti  #16 'cancers, gastric':ab,ti  #17 'gastric cancers':ab,ti  #18 'stomach cancer':ab,ti  #19 'cancer, stomach':ab,ti  #20 'cancers, stomach':ab,ti  #21 'cancer of the stomach':ab,ti  #22 'gastric cancer, familial diffuse':ab,ti  #23 #2 OR #4 OR #5 OR #6 OR #7 OR #8 OR #9 OR #10 OR #11 OR #12 OR #13 OR #14 OR #15 OR #16 OR #17 OR #18 OR #19 OR #20 OR #21 OR #22  #24 'random':ab,ti  #25 'placebo':ab,ti  #26 'double-blind':ab,ti  #27 #24 OR #25 OR #26  #28 #23 AND #27 |
